# Weighted gene co-expression network analysis reveals key biomarkers and immune infiltration characteristics for bronchial epithelial cells from asthmatic patients

**DOI:** 10.1097/MD.0000000000037796

**Published:** 2024-04-19

**Authors:** Qianqian Liu, Xiaoli Tang, Haipeng Xu, Jie Wen, Yangyang Chen, Shoubin Xue

**Affiliations:** a Respiratory Department, The First People’s Hospital of Lanzhou City, Lanzhou, Gansu, China; b Traditional Chinese Medical Hospital of Xinjiang Uygur Autonomous Region, Xinjiang, China; c The Third Clinical Medical College, Zhejiang Chinese Medical University, Hangzhou, China; d Xinjiang Gem Flower Hospital, Xinjiang, China.

**Keywords:** asthma, immune cell infiltration, WGCNA

## Abstract

**Background:**

Asthma ranks among the most prevalent non-communicable diseases worldwide. Previous studies have elucidated the significant role of the immune system in its pathophysiology. Nevertheless, the immune-related mechanisms underlying asthma are complex and still inadequately understood. Thus, our objective was to investigate novel key biomarkers and immune infiltration characteristics associated with asthma by employing integrated bioinformatics tools.

**Methods:**

In this study, we conducted a weighted gene co-expression network analysis (WGCNA) to identify key modules and genes potentially implicated in asthma. Functional annotation of these key modules and genes was carried out through gene ontology (GO) and Kyoto Encyclopedia of Genes and Genomes (KEGG) analysis. Additionally, we constructed a protein–protein interaction (PPI) network using the STRING database to identify 10 hub genes. Furthermore, we evaluated the relative proportion of immune cells in bronchial epithelial cell samples from 20 healthy individuals and 88 asthmatic patients using CIBERSORT. Finally, we validated the hub genes and explored their correlation with immune infiltration.

**Results:**

Furthermore, 20 gene expression modules and 10 hub genes were identified herein. Among them, complement component 3 (C3), prostaglandin I2 receptor (PTGIR), parathyroid hormone-like hormone (PTHLH), and C-X3-C motif chemokine ligand 1 (CX3CL1) were closely correlated with the infiltration of immune cells. They may be novel candidate biomarkers or therapeutic targets for asthma. Furthermore, B cells memory, and plasma cells might play an important role in immune cell infiltration after asthma.

**Conclusions:**

C3, PTGIR, CX3CL1, and PTHLH have important clinical diagnostic values and are correlated with infiltration of multiple immune cell types in asthma. These hub genes, B cells memory, and plasma cells may become important biological targets for therapeutic asthma drug screening and drug design.

## 1. Introduction

Asthma is one of the most common chronic and non-communicable diseases in children and adults. It affects an estimated 314 million individuals globally, posing a heavy burden to families and society.^[1,2]^ The disease is typically characterized by symptoms such as cough, dyspnea, chest tightness, and the appearance of pathognomonic features dominated by airflow obstruction, bronchial hyperresponsiveness, and airway inflammation.^[3]^ Previous studies have exposed that^[4]^ during the development of asthma, cellular inflammatory response plays an instrumental role. Therefore, exploring the distribution patterns of relevant gene modules and immune cell subtypes in asthmatic patients is conducive to the diagnosis and treatment of asthma.

Weighted gene co-expression network (WGCNA), an important biological method of analyzing molecular mechanisms and network relationships, is widely used to analyze a large amount of gene expression data.^[5]^ WGCNA is typically employed to detect co-expressed gene modules and explore co-expression module gene associations.^[6]^ In the present study, we constructed a co-expression network by WGCNA using microarrays of asthma and healthy patients (GSE43696) in an attempt to identify asthma-related therapeutic targets from highly correlated gene modules.

CIBERSORT is a deconvolution algorithm that analyzes the subtype of each immune cell, accurately quantifying different immune cell components.^[5]^ In the present study, the relative proportion of immune cells in bronchial epithelial cell samples from 20 healthy persons and 88 asthmatic patients was assessed using CIBERSORT. The acquired immune cell profiles provided the proportion and activation states of the 22 immune cell subtypes. In conclusion, we constructed co-expression modules by analyzing the expression data of asthmatic and healthy individuals and performed an integrated bioinformatics analysis of the modules of interest in the hope of identifying novel potential biomarkers for the diagnosis and treatment of asthma.

## 2. Materials and methods

### 2.1. Dataset information

In our analysis, we have leveraged gene expression datasets obtained from the gene expression omnibus repository, focusing on 3 distinct cohorts: the GSE43696 training set with a composition of 88 asthmatic patients and 20 controls, the GSE63142 validation set with 128 asthmatic patients and 27 controls, and the GSE64913 validation set with 28 asthmatic patients and 42 controls.^[7]^ The GSE43696 dataset encompasses tissue samples from 50 individuals diagnosed with mild to moderate asthma and 38 with severe asthma. The GSE63142 set includes samples from 72 patients with mild to moderate asthma and 56 with severe asthma, while the GSE64913 set features samples from 28 patients with severe asthma. This comprehensive approach ensures a robust and multifaceted evaluation of the gene expression patterns associated with asthma severity.

### 2.2. Construction of the co-expression network

We identified co-expression gene modules using the “WGCNA” package in R and analyzed and visualized the strength of interaction between modules with the Heatmap toolkit.^[8]^ The GS (gene significance) and MM (module membership) metrics were utilized to estimate module trait associations (the quantitative metric of mm was defined as the correlation of module with gene expression profiles, while GS was defined as the absolute value of the correlation between genes and traits), and the corresponding module gene information was extracted for subsequent analyses.

### 2.3. Functional enrichment analysis

The differential genes of the target modules were extracted from the network, and enrichment analysis was performed, leading to the exploration of the functions of the modules. Enrichment criteria for gene ontology (GO) terms and Kyoto Encyclopedia of genes and genomes (KEGG) pathways were *P* < .05 and enriched gene count > 2.

### 2.4. Immune infiltration was analyzed by CIBERSORT

CIBERSORT is a deconvolution algorithm that translates a normalized gene expression matrix into components of infiltrating immune cells. In this study, an immune cell assessment of the infiltrates in asthma was conducted on datasets GSE43696 and GSE64913. The 22 types of infiltrated immune cells consisted of B cells (naïve and memory B cells), T cells (CD8+, naïve CD4+, memory resting CD4+, memory activated CD4+, follicular helper, regulatory, and γδ T cells), NK cells (resting and activated NK cells), monocytes, macrophages (M0 macrophages, M1 macrophages, and M2 macrophages), dendritic cells (resting and activated dendritic cells), mast cells (resting and activated mast cells), eosinophils and neutrophils.

### 2.5. Protein-protein interaction (PPI) network analysis

To explore the interrelationship between proteins encoded by different genes, the genes were imported into the string website for the ensuing analysis, with a minimum interaction score of more than 0.4, and isolated nodes in the network removed. The results were then saved into TSV format files for visual analysis with the Cytoscape software (version: 3.7.1).^[9]^ Cytohubba is a plugin downloaded from Cytoscape Appstore, which was applied to detect core genes in the PPI network with default parameters.

### 2.6. ROC curve analysis and Immune infiltration analyses

ROC curve analysis were performed to validate the diagnostic accuracy of the biomarkers. We used the R package “pROC” to conduct ROC analysis. we employed the R packages (“tidyverse”) to analyze the correlation between these hub genes and 22 immune cells, which have got via the CIBERSORT.

## 3. Results

### 3.1. Workflow

**Figure 1.**
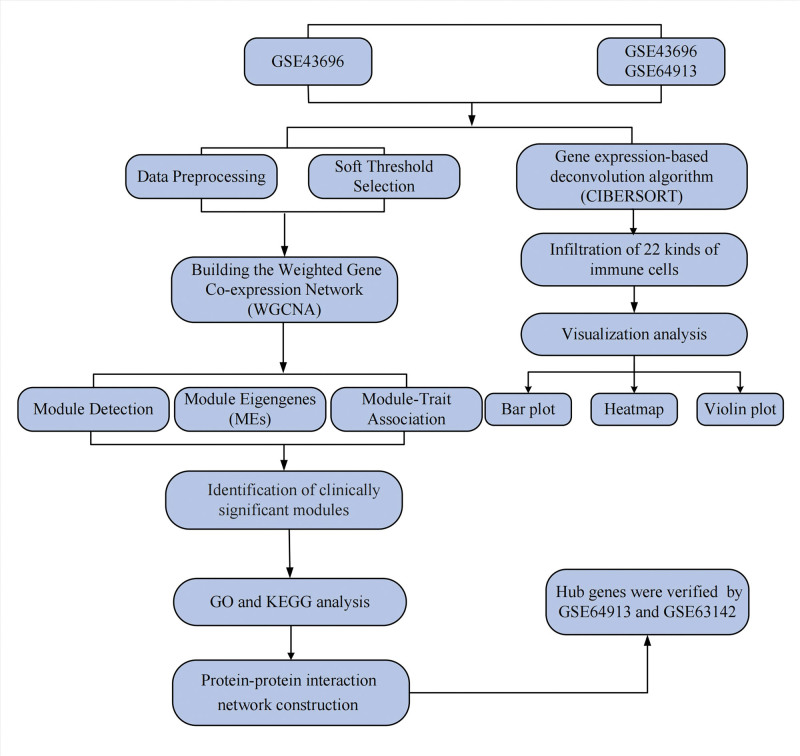
Workflow diagram.

A schematic of the workflow is illustrated in Figure 1. We constructed a co-expression network in samples of asthma patients and samples of healthy people and identified several modules of clinical significance. The function of the key modules was then analyzed to identify the core differential genes in asthma patients.

### 3.2. Construction of weighted gene co-expression network

**Figure 2.**
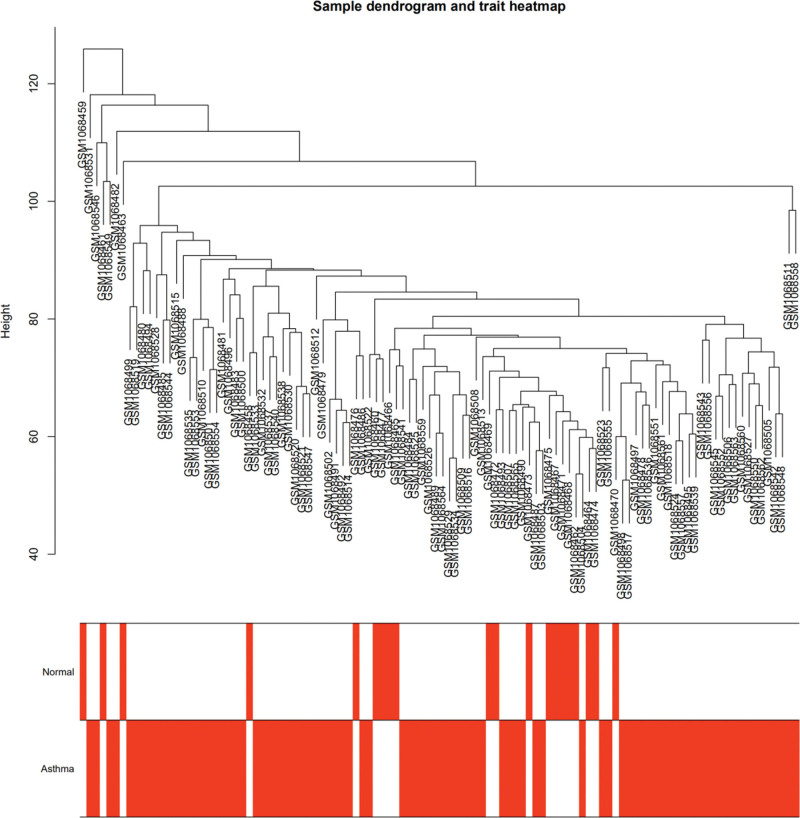
A clustering dendrogram of the 108 individual samples, with the clustering tree reflecting the distance between the samples.

**Figure 3.**
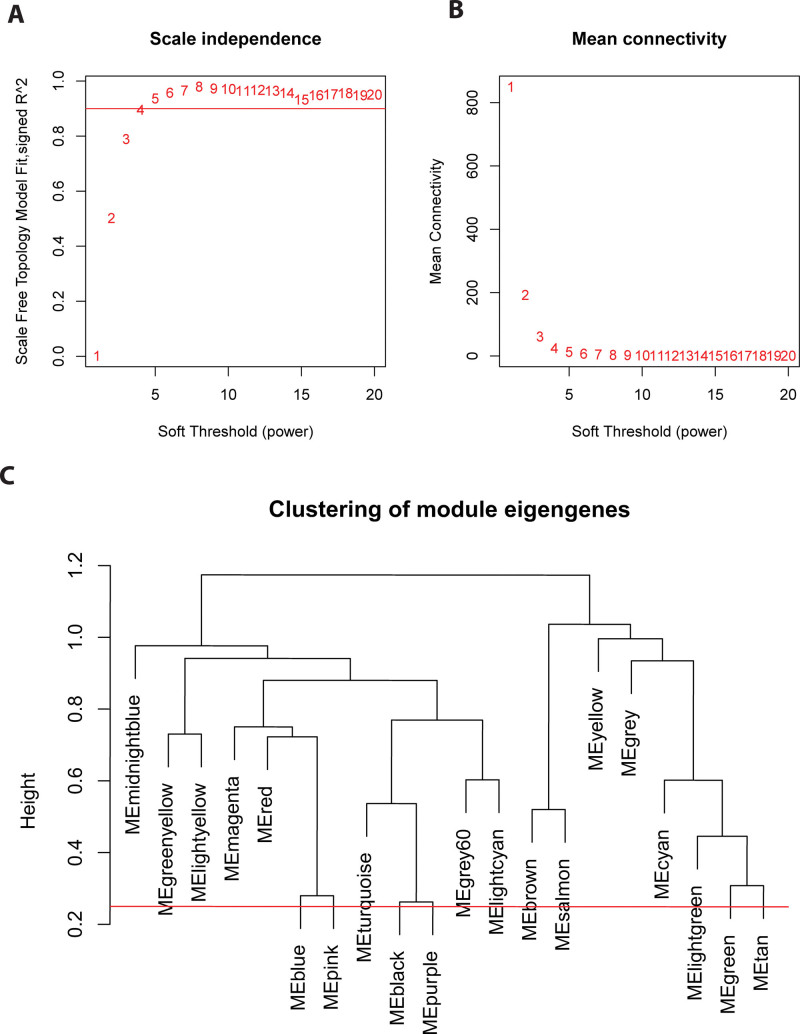
Analysis of network topology for various soft-thresholding powers. (A) The x-axis represents the soft threshold power, while the y-axis represents the scale topological model fitting index (B) the x-axis reflects the soft threshold power, while the y-axis reflects the average connectivity (C) the sample module partition and the clustering tree constructed by module characteristic genes can reflect the distance between the modules. The modules with a smaller distance between them (below the red line) were consolidated into 1 module, and finally, 20 modules were identified.

To identify key genes associated with asthma, WGCNA was applied to process the GSE43696 dataset. Firstly, the samples were clustered by Pearson correlation coefficient, and after removing evident outlier samples, a sample cluster tree was constructed (Fig. 2). Subsequently, suitable soft thresholds were explored, and the fit was determined to be sufficiently high and the average connectivity relatively high when R^2^ > 0.9 (Fig. 3A and B). Finally, the threshold was set to 0.25, and 20 modules were identified by utilizing the wgcnar package (Fig. 3C).

### 3.3. Identification of clinically significant modules

**Figure 4.**
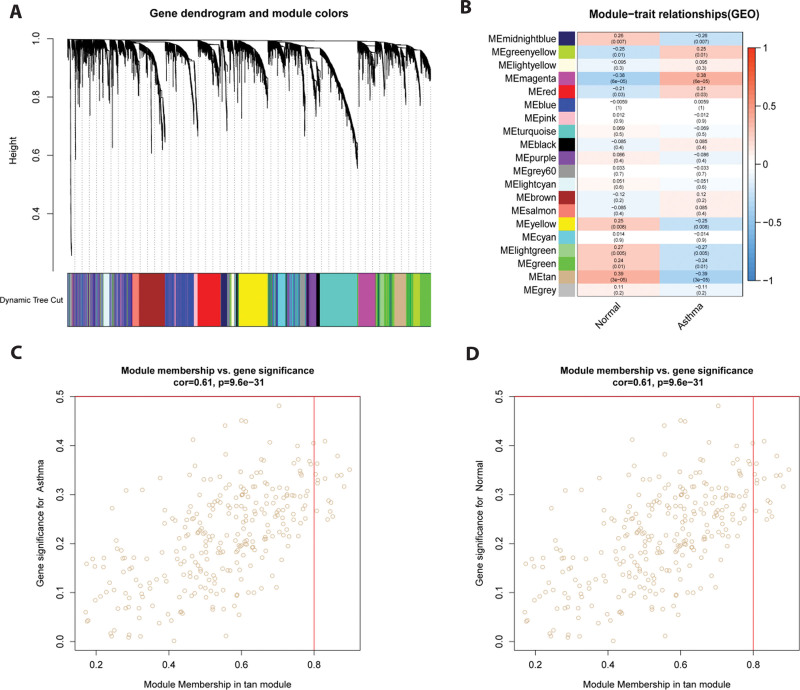
To identify key modules associated with asthma patients, a hierarchical cluster analysis was performed. (A) Cluster tree plots and module feature relationship plots were utilized to detect co-expression clusters with the corresponding color distribution. (B) Modular trait associations were assessed by correlations between me and sample traits. Each row represents a feature eigenmodule, with darker red representing a stronger positive correlation and darker blue representing a negative correlation. (C-D) Scatter plot of gene significance (GS) of asthma severity versus module membership (mm) in the tan module; asthmatic group on the left, and normal group on the right.

Correlation analysis of gene expression and disease signatures were carried out across all samples using WGCNA, thereby constructing 20 gene expression modules (Fig. 4A). Next, we correlated the modules with clinical features and searched for the most significant modules (Fig. 4B), and uncovered that the gene expression of the tan module was most closely related to the 2 groups (normal and asthma groups) (Fig. 4C and D). Therefore, the tan module was chosen for subsequent analysis.

### 3.4. Functional analysis of the key module

**Figure 5.**
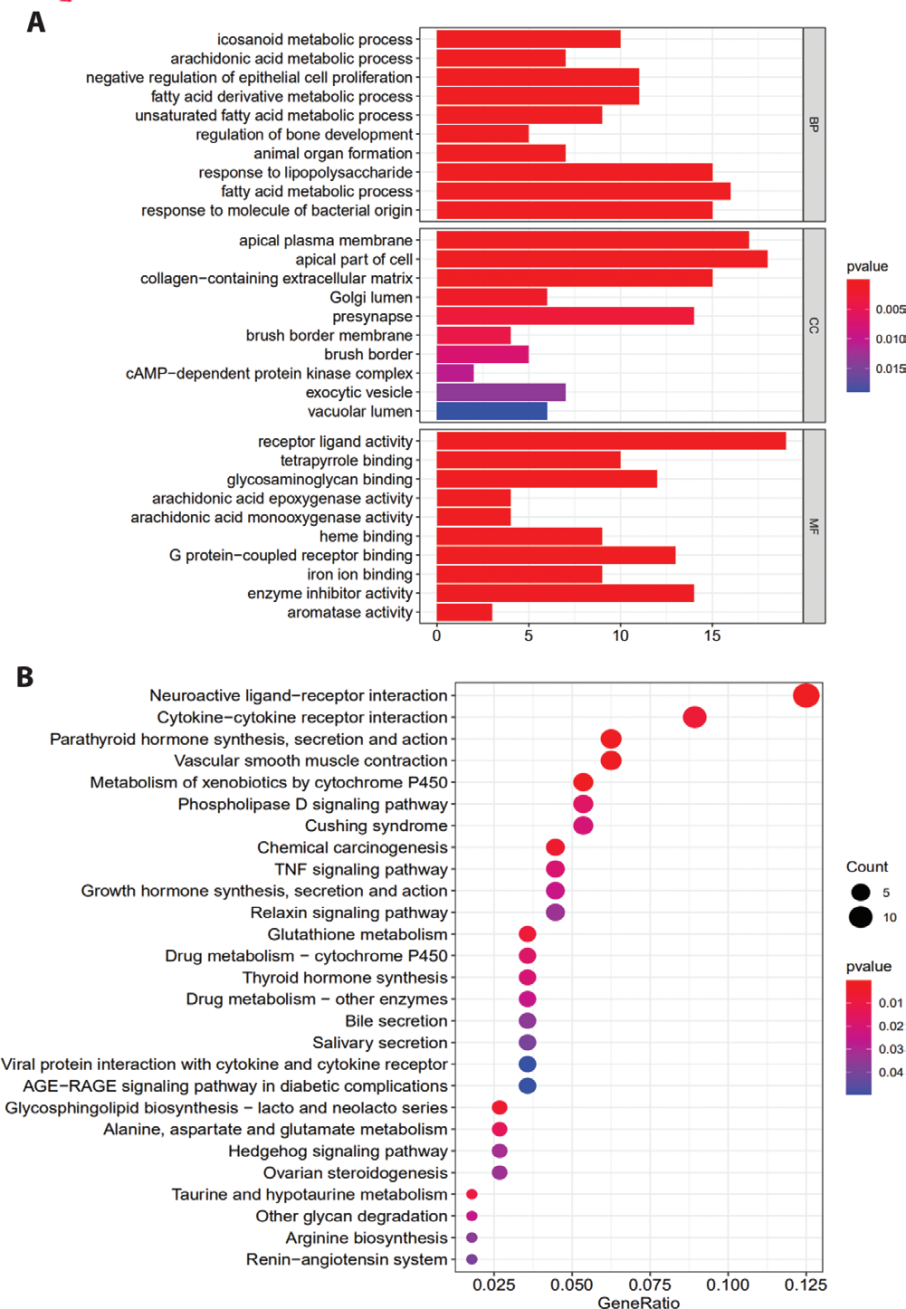
Functional Analysis of the Key Module: (A) Gene Ontology (GO) enrichment analysis of genes involved in the tan module. (B) Enriched pathways of genes in the tan module by the Kyoto Encyclopedia of Genes and Genomes (KEGG).

GO and KEGG analyses of the tan module were conducted, and the results of the GO analysis demonstrated that the genes in the tan module were predominantly related to processes including response to molecules of bacterial origin, response to lipopolysaccharide, and fatty acid metabolism. The cellular components were enriched in apical plasma membrane, including apical part of cells and collagen-containing extracellular matrix. Lastly, molecular functions (MF) were mainly enriched in receptor-like activity, G protein-coupled receptor binding, etc (Fig. 5A). Thereafter, KEGG analysis of the genes was performed for the tan module to identify the pathways regulated by the module. The analysis revealed that the regulatory pathways of the tan module were mainly related to neuroactive ligand-receptor interactions, cytokine receptor interactions, and vascular smooth muscle contact (Fig. 5B).

### 3.5. Immune infiltration analyses

**Figure 6.**
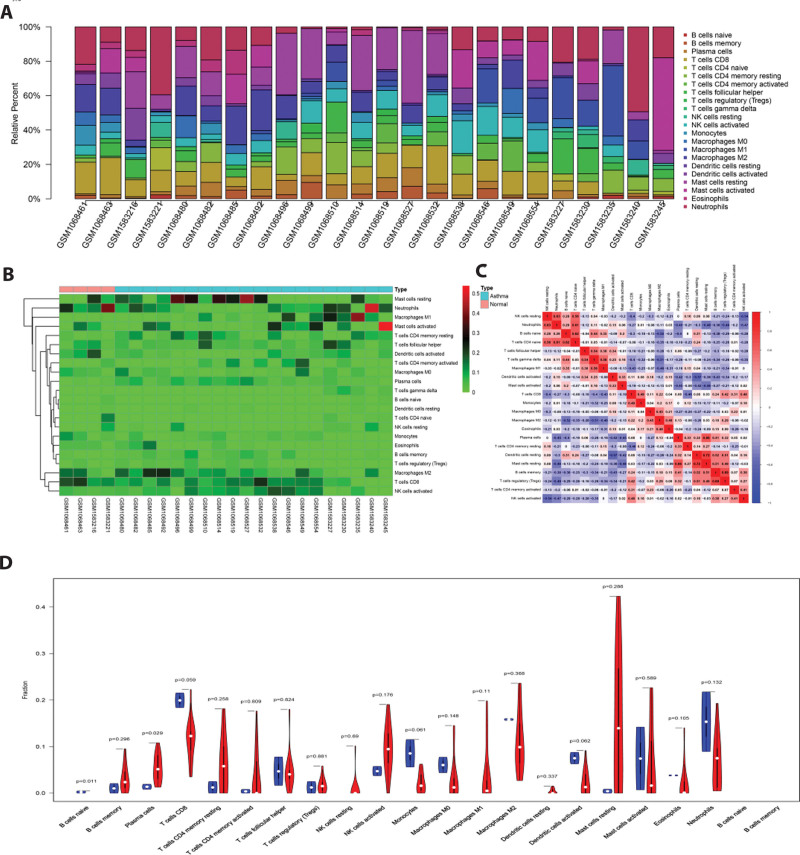
Immune infiltration of bronchial epithelial cell samples from asthmatic patients versus healthy individuals. (A) Percentage distribution histograms of the 22 immune cell subtypes. (B) Heat map of the 22 immune cell subtypes. (C) Heat map of the proportion of the 22 immune cell subtypes in each sample. (D) Violin plots of the differences in immune cell infiltration between asthmatics and healthy individuals.

Based on the above, CIBERSORT was used to calculate each sample and screen all samples with *P* < .05 as the threshold. Then, the difference in immune infiltration among the 22 immune cell subsets was analyzed. The histogram delineates the distribution of various immune cells in asthma patients and healthy controls. The results showed that naïve B cells, memory B cells, macrophages M1, macrophages M2, resting mast cells, and plasma cells were the main immune infiltrating cells (Fig. 6A and B). As depicted in Figure 6D, compared with healthy tissues, the tissues of asthmatic patients contained a lower proportion of naïve B cells (*P* = .011). In contrast, the proportion of plasma cells was relatively higher (*P* = .029). Additionally, there was a correlation between the proportion of different infiltrating immune cell subsets in healthy and asthma samples. For example, the correlation coefficient between resting mast cells and dendritic cells rest was 0.83, whereas that between neutrophils and resting dendritic cells was −0.68 (Fig. 6C).

### 3.6. Construction of the protein-protein interaction network

**Figure 7.**
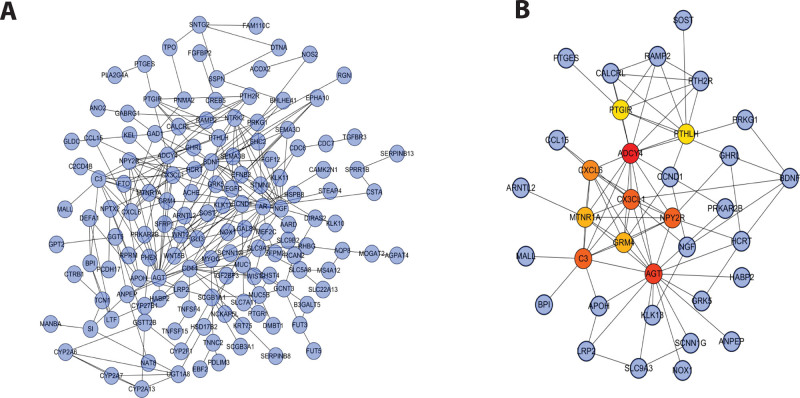
Construction of a protein-protein interaction network. (A) Protein-protein interaction (PPI) networks differentially associated with asthma patients. (B) Core gene cluster in the co-expression network with a total of 10 core genes. The depth of the color reflects the level of core genes, which ranges from low to high.

To investigate the pathogenesis of asthma in a near step and prospective biomarkers for the potential treatment of asthma, a PPI network was constructed with the string database for the clinically meaningful module (tan module) (Fig. 7A). The cytohubba tool was applied to screen and visualize the 10 most significant hub genes in the network, as displayed in Figure 7B. The results showed that the 10 most significant hub genes in the network were complement component 3 (C3), Angiotensinogen (AGT), Melatonin Receptor 1A (MTNR1A), C-X-C Motif Chemokine Ligand 6 (CXCL6), PTGIR, Adenylate Cyclase 4 (ADCY4), CX3CL1, Glutamate Metabotropic Receptor 4 (GRM4), PTHLH, and Neuropeptide Y Receptor Y2 (NPY2R).

### 3.7. Validation of hub genes and correlation with immune infiltration

**Figure 8.**
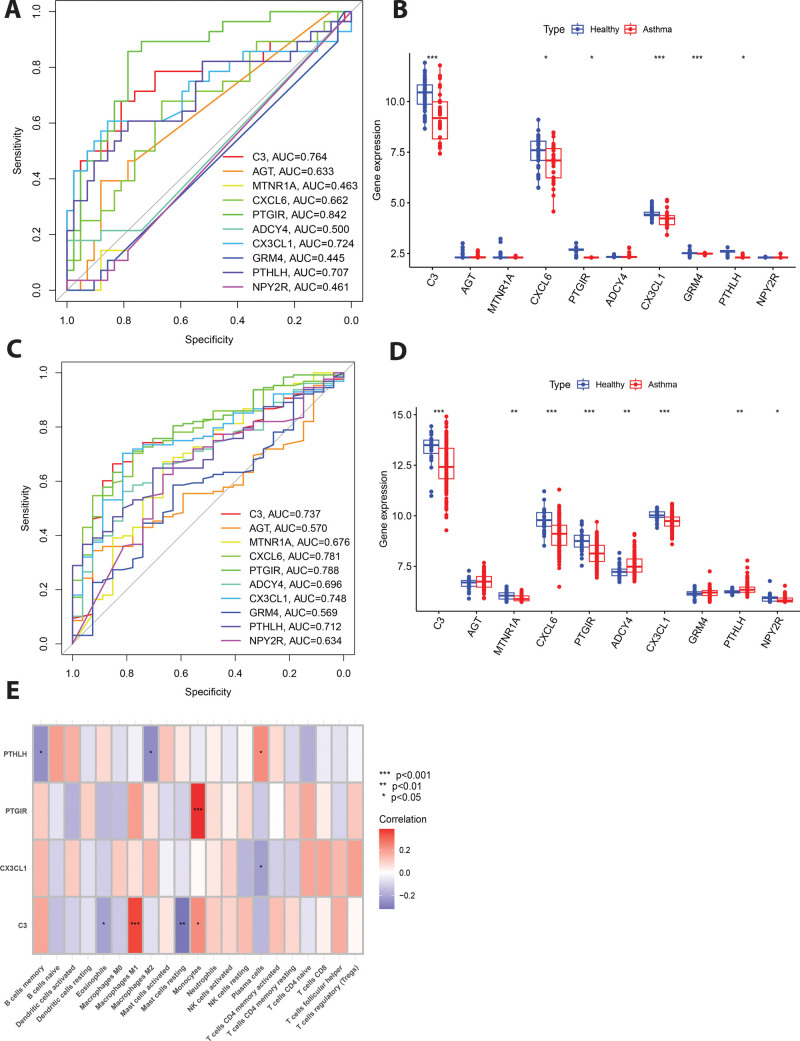
Validation of hub genes and correlation with immune infiltration. (A) Validation of candidate hub genes via ROC curve analysis. The 4 hub genes with an AUC higher than 0.70 were considered hub genes of asthma in GSE64913 dataset. (B) Differential expression levels of the 10 hub genes in GSE64913 dataset. (C) Validation of candidate hub genes via ROC curve analysis. The 4 hub genes with an AUC higher than 0.70 were considered hub genes of asthma in GSE63142 dataset. (D) Correlation analysis of 4 hub genes and immune cell infiltration. Red represents positive correlation whereas blue represents negative correlation.

The validation dataset (GSE64913 and GSE63142) were retrieved from the gene expression omnibus database. ROC curves were then used to analyze the diagnostic value of the 10 hub genes in asthma. Four of the 10 genes (C3, PTGIR, CX3CL1, and PTHLH) were considered to have diagnostic value, given their AUC was higher than 0.70, implying that they may be used to diagnose asthma patients with high specificity and sensitivity (Fig. 8A and C). In our study, we conducted a comparative analysis of the expression levels of 10 hub genes within bronchial epithelial cells from asthmatic patients and healthy controls (Fig. 8B and D). Our findings revealed that the expression of C3, CX3CL1, GRM4, CXCL6, PTGIR, and PTHLH was notably reduced in the bronchial epithelial cells of asthmatic subjects when compared to those from healthy individuals, with statistical significance (*P* < .05).

To further validate our findings, we analyzed the correlation between 4 hub genes (C3, PTGIR, CX3CL1, and PTHLH) and immune cell infiltration in asthma, This result indicates that C3, PTGIR, CX3CL1, and PTHLH expression were significantly correlated with the infiltration of immune cells (Fig. 8E). For example, the C3 expression significantly correlated with 4 immune cell types phages M1、Mast cells resting and monocytes (*P* < .05). PTHLH expression significantly correlated with 3 immune cell types (*P* < .05).

## 4. Discussion

Asthma is the most common chronic respiratory disease worldwide and is characterized by high morbidity and mortality. In the US, the annual asthma-related treatment costs are as high as $80 billion.^[10,11]^ Earlier studies have demonstrated that^[12]^ patients with different types of asthma have varying risk profiles with respect to COVID-19 infection. Therefore, mechanistic studies are instrumental to enhance asthma treatment and improve the prognosis of asthma patients. In this study, WGCNA and CIBERSORT algorithms were employed to explore the pathological processes and core genes of bronchial epithelial cell samples from asthma patients. First, we conducted a weighted gene network construction by subjecting the downloaded data. The module with the most significant clinical significance (tan module) was then subjected to GO and KEGG analyses. The results of the KEGG analysis of the tan module indicate that the tan module were mainly related to neuroactive ligand-receptor interactions, cytokine receptor interactions, and vascular smooth muscle contact. Neuroactive ligand-receptor interactions are pivotal in the pathophysiology of asthma, where neurotransmitters and neuropeptides, such as acetylcholine and histamine, bind to receptors on airway smooth muscle cells. This binding triggers airway smooth muscle contraction, intensifying asthma symptoms. Neuropeptides including substance P and nerve growth factor further modulate airway inflammation and ASM cell proliferation.^[13]^ Cytokines, upon binding to their respective cell surface receptors, initiate signaling cascades that facilitate the recruitment and activation of inflammatory cells, as well as influence ASM cell function by promoting contraction and proliferation, contributing to airway constriction and remodeling. Excessive vascular smooth muscle contraction is a primary factor in airway narrowing and respiratory distress in asthma, while ASM cell hyperplasia and remodeling are hallmarks of the disease airway changes. A thorough understanding of these complex interactions is essential for the development of innovative therapeutic approaches to asthma.^[14]^ Thereafter, the samples were subjected to immune infiltration analysis; the results demonstrated that tissues from asthma individuals contained a lower proportion of naïve B cells and a relatively higher proportion of memory B cells and plasma cells. Subsequently, a protein-protein interaction network was constructed, and the 10 core genes (C3, AGT, MTNR1A, CXCL6, PTGIR, ADCY4, CX3CL1, GRM4, PTHLH, and NPY2R) were identified. Finally, ROC curves were used to analyze the diagnostic value of the 10 hub genes in asthma. Among them, 4 genes validated by ROC curve analysis with AUC > 0.70 revealed potential diagnostic values for asthma, including C3, PTGIR, CX3CL1 and PTHLH. Interestingly, all 4 genes are deeply involved in the infiltration of a variety of immune cell types.

Innate immunity plays a critical role in the initiation and progression of asthma. Complement C3 is a serum protein involved in humoral immune and acute responses. Activated C3 exerts inflammatory mediator effects, bactericidal effects, immune effects, etc.^[15]^ The complement component C3 is a key player in the immune response, particularly in adaptive immunity. It is involved in phagocytosis, respiratory burst, and airway inflammation.^[16]^ This complement component is involved in the activation of the immune response and can promote the recruitment of immune cells to sites of inflammation. It is particularly known for its role in the opsonization of pathogens, enhancing phagocytosis by macrophages and neutrophils.^[17]^ Prior studies have found that C3 possesses numerous proinflammatory and immunomodulatory properties and is essential for the development and regulation of asthma. Higher complement C3 concentrations were correlated with increased asthma hospitalizations in adults and a higher likelihood of asthma exacerbations in allergic asthma patients.^[18]^ Our investigation has revealed a notable reduction in C3 expression among individuals with asthma. This downregulation of C3 in asthmatic patients may have significant implications for the immune system ability to clear pathogens and resolve inflammatory responses effectively. The diminished levels of C3 could potentially impair the complement system capacity to opsonize pathogens, thereby affecting the phagocytic activity of immune cells and the overall inflammatory resolution process. This finding underscores the importance of further research to elucidate the role of C3 in the pathophysiology of asthma and its potential as a therapeutic target.

Chemokines are highly implicated in mediating the development and maintenance of asthma.^[19]^ CX3CL1 is the first member of a new chemokine family abundant in endothelial cells, epithelial cells, lymphocytes, neurons, glial cells, and osteoblasts. Its expression in endothelial and epithelial cells is of great significance.^[20]^ Studies have reported that CX3CL1 can bind to the CX3CL1 receptor. A previous study exposed that the administration of a CX3CL1 antagonist to antigen-sensitive mice promoted the survival of Th2 cells in the inflamed airway and suppressed airway inflammation.^[21]^ Our current results demonstrate that CXC3L1 expression is correlated with infiltration of multiple immune cell types in asthma. It might be a potential therapeutic target for asthma patients.

In addition, a huge body of evidence shows that interfering with chemokines or chemokine receptors is a new approach to the treatment of asthma. For instance, studies have evinced that rhinovirus infection in asthma patients leads to increased release of CXCL8/IL-8, while inhibition of CXCL8 expression can delay the exacerbation of asthma symptoms induced by viruses. Moreover, up-regulation of the anti-inflammatory chemokine CXCLl17 can limit airway eosinophilic inflammation.^[22,23]^ However, the relationship between CXCL6 and asthma remains elusive.

MTNR1A is a 7-transmembrane protein belonging to the GPCR superfamily and plays an important role in regulating the central nervous and immune systems in mammals.^[24]^ Chloe Sarnowski et al performed a series of genetic analyses of asthma and observed that methylation within MTNR1A gene mediated the effect of paternally-inherited variants on the comorbidity of asthma and allergic rhinitis.^[25,26]^ MTNR1A is involved in the regulation of circadian rhythms and sleep-wake cycles. Melatonin has anti-inflammatory properties, and its receptors may modulate the immune response, which could be relevant to asthma.^[27]^ PTGIR is involved in the regulation of inflammatory responses. It is expressed on various immune cells, including T cells, B cells, and macrophages. The modulation of PTGIR expression could influence the balance between proinflammatory and anti-inflammatory responses, affecting the overall immune cell activity in the airways.^[28]^ PTGIR is highly implicated in the modulation of vascular homeostasis and airway inflammatory diseases,^[29]^ considering that it plays a key role within the immune system, ptgir agonists have been reported to exert anti-inflammatory and antiviral effects in preclinical models of asthma and COPD.

Our study also identified several highly-expressed but poorly-studied core genes in asthma. For example, GRM4 belongs to the GRM protein family and plays an important role in the regulation of ion channels, neuronal excitability, and neurotransmitter release,^[30]^ Indeed, overexpression of GRM4 can significantly inhibit the proliferative, migratory, and invasive abilities of tumor cells, while GRM4 can also suppress osteosarcoma growth through a non-cell-autonomous mechanism by regulating IL23.^[31]^ Its role in asthma may involve neuroinflammatory aspects of the disease. The NPY2R is involved in stress responses and appetite regulation. It may play a role in the neuroendocrine aspects of asthma, potentially influencing disease severity and response to stress.^[32]^ ADCY4 is involved in the immunoregulation of cAMP by inhibiting caspase-11 inflammasome activation through mediated cAMP synthesis.^[33]^

As is well documented, genetics and environment are important factors in asthma pathogenesis. Rare variants in the AGT gene are a major cause of asthma susceptibility in African Americans.^[34]^ PTHLH is a gastrin-regulated growth factor that aids in maintaining gastric epithelial homeostasis and also promotes the epithelial mesenchymal transition of intestinal epithelial cells.^[35,36]^ Interestingly, some scholars have postulated^[37]^ that the intestine is a key site for the development of immune cells, and studying the interactions between the gut microbiota and immune cells can provide potential targets for the treatment of asthma. Most importantly, immune cell infiltration analysis revealed that PTHLH was closely related to immune cell infiltration in asthmatic patients. Therefore, we hypothesize that PTHLH may be a key target involved in the intestinal immune asthma link. Our research underscores the imperative for further exploration into the molecular mechanisms underlying the role of the core genes identified in asthma pathogenesis, including the complex signaling networks and interactions they form with other immune and inflammatory components. Future endeavors should focus on clinical trials to assess the potential of these genes as therapeutic targets, with an emphasis on the development of targeted therapies and rigorous evaluation of their clinical efficacy and safety profiles.

The study acknowledges certain limitations. The functional validation of the identified genes and their potential to serve as biomarkers or therapeutic targets demands additional experimental confirmation, encompassing both in vitro and in vivo research. Given the multifaceted nature of asthma, a multidisciplinary approach is imperative, integrating genetic, immunological, and environmental research to gain a comprehensive understanding of the disease etiology and to devise effective treatment modalities.

## 5. Conclusions

In conclusion, our study investigated asthma microarray samples by WGCNA and immune infiltration. In this study, we identified 20 gene expression modules and 10 hub genes. Among them, C3, PTGIR, CX3CL1, and PTHLH has important clinical diagnostic value and are correlated with infiltration of multiple immune cell types in asthma. The 4 genes may be novel candidate biomarkers or therapeutic targets which have not been previously associated with asthma. Lastly, memory B cells and plasma cells may become important biological targets for therapeutic asthma drug screening and design.

## Acknowledgments

We thank all teachers and students there for directing empirical methods. We thank Freescience (Contact Method: freescience@zju.edu.cn) for the help in language polishing.

## Author contributions

**Conceptualization:** Qianqian Liu, Yangyang Chen.

**Data curation:** Qianqian Liu, Yangyang Chen.

**Formal analysis:** Qianqian Liu, Xiaoli Tang, Yangyang Chen.

**Funding acquisition:** Qianqian Liu, Xiaoli Tang.

**Investigation:** Haipeng Xu.

**Methodology:** Haipeng Xu.

**Project administration:** Haipeng Xu.

**Software:** Jie Wen.

**Supervision:** Jie Wen.

**Validation:** Qianqian Liu, Jie Wen.

**Visualization:** Shoubin Xue.

**Writing – original draft:** Shoubin Xue.

**Writing – review & editing:** Shoubin Xue.
